# Study on the Influence of Furfural on the Analysis of SARA Fractions

**DOI:** 10.3390/molecules31193409

**Published:** 2026-09-24

**Authors:** Hao Song, Zhi Shen, Xizhou Shen, Qiang Ren, Han Zhou

**Affiliations:** 1 Key Laboratory for Green Chemical Process of Ministry of Education, School of Chemical Engineering & Pharmacy, Wuhan Institute of Technology, Wuhan 430073, China; 2 Sinopec Research Institute of Petroleum Processing, Beijing 100083, China

**Keywords:** heavy oils, furfural extraction, intermolecular interactions, molecular simulation, vacuum-oven method

## Abstract

Elution chromatography was used to analyze the content of SARA (saturate, aromatic, resin, and asphaltene) fractions in simulated raffinate and extract phases containing furfural (FUL), and the influence mechanism of furfural on the content analysis of SARA in the two phases was explored by molecular simulation. The experimental results show that the analysis of SARA fractions was more affected by the presence of a higher content of furfural in the sample. During the process of sample loading, some chromatographic bands of resins and aromatics significantly shifted downward: some aromatics were eluted earlier when saturates were eluted with n-heptane, and some resins were eluted earlier when aromatics were eluted with toluene. In addition, the molecular simulation results indicate that the ability of the model molecules and FUL to adsorb onto the surface of FCP-Al_2_O_3_ followed the order: saturates < aromatics < FUL < resins. Additionally, the interaction strength among the saturate, aromatic, resin model molecules and FUL was ranked as follows: saturates < aromatics < resins. The molecular simulation results reveal the mechanism by which furfural influences the analysis of SARA fractions: unadsorbed furfural promotes the desorption of some aromatics and resins, resulting in the downward movement of the chromatographic bands and affecting the elution effect, thereby affecting the accuracy of SARA fraction analysis data. Furthermore, the improved vacuum distillation method, namely, the vacuum-oven method, not only has good precision (RSD < 1%) and accuracy (recovery: 99.7–100.5%) in the determination of furfural content in simulated raffinate- and extract-phase samples but also has good precision (RSD < 1%) on real raffinate- and extract-phase samples under optimized conditions.

## 1. Introduction

With the increasing shortage of petroleum resources in today’s world, crude oil has become heavy and inferior [1,2,3]. Faced with the gradual growth of the heavy crude oil proportion, the full exploitation and rational processing of heavy oil have become a concern for researchers in consequence. Currently, the processing technologies for heavy oil mainly involve hydrogenation [1,4,5,6,7] and carbon rejection [5,6,8,9,10,11]. As one of the important decarburization processes, catalytic cracking has problems such as high coke yield and serious catalyst poisoning caused by sulfur, nitrogen heteroatoms or heavy metals in heavy oil processing [12,13,14,15]. Therefore, it is necessary to pretreat the heavy oil feedstock before it enters the fluid catalytic cracking (FCC) unit.

Solvent extraction, as a prominent separation method in the refining industry, has been widely used in residual oil deasphalting [16,17,18], lubricating oil refining [19,20], reforming oil extraction of aromatics [21,22,23,24] and so on. Unsaturated fractions (i.e., aromatics, resins, and asphaltenes) in vacuum residue are removed by a combination of solvent deasphalting (SDA) and furfural extraction, and the obtained saturate fractions can be used as high-quality catalytic cracking raw materials. In the process of furfural extraction for deasphalted oil (DAO), the removal rates and selectivity of unsaturated fractions in DAO are taken as key indexes, which are directly related to extraction efficiency, solvent–oil ratio and energy consumption. It is noteworthy that the accurate analysis of SARA (saturate, aromatic, resin, and asphaltene) fractions in raffinate and extract oil, as well as furfural, in both the raffinate phase and the extract phase is an important prerequisite for obtaining extraction efficiency and selectivity.

For the analysis of SARA fractions, there are currently many determination methods [25,26,27,28,29,30], such as elution chromatography (EC) [25,26], high-performance liquid chromatography (HPLC) [27], thin-layer chromatography with flame ionization detector (TLC-FID) [28], etc. However, the solvent must be removed before analyzing the four components by the aforementioned methods [31], as this is likely attributable to the interaction between polar solvents (e.g., furfural, NMP, and phenol) and SARAs. The interaction may hinder the analysis of SARAs in heavy oils, yet the extent of this interference and its underlying mechanism remain unexamined. To address these concerns, the commonly employed technique of elution chromatography was utilized to analyze SARAs in simulated raffinate and extract phases containing furfural. Furthermore, the influence of furfural on the analysis of SARA contents in both phases was investigated through molecular simulations. As furfural may impact the analysis of SARA contents, it is crucial to precisely determine its content. An improved vacuum distillation method, namely, the vacuum-oven method, was employed to determine furfural concentration in raffinate phase and extract phase.

## 2. Experimental Methods

### 2.1. Materials

Deasphalted oil (DAO) was obtained from China Petroleum Chemical Co., Jiujiang branch (Jiujiang, China). Heavy paraffin oil (HPO), furfural (FUL; AR, 99%), and silica gel self-indicator were purchased from Shanghai Macklin Biochemical Technology Co., Ltd (Shanghai, China). Heptane (AR, 99%) and aluminum oxide for chromatography purposes (FCP-Al_2_O_3_) were obtained from Sinopharm Group (Qingdao, China). Toluene (AR, 99.5%) was provided by Xilong Scientific (Sichuan, China). The properties of DAO and HPO are listed in Table 1.

### 2.2. Sample Preparation

DAO and HPO (mass ratio of 1:3) were poured into a beaker and vigorously stirred at 100 °C for 1 h, and then the mixture was left to cool to room temperature to obtain DAO-HPO mixed oil. FUL and DAO-HPO were mixed well by an adjustable high-speed homogenizer (FSH-2A, Zhengzhou Shengke Experimental Instruments Co., Ltd, Zhengzhou, China) equipped with a homogenizer knife (L120 × Ф17.5 mm). DAO-HPO samples containing FUL mass fractions of 5%, 15%, 20%, 25%, 30%, 80%, 85%, 90% and 95% were obtained and denoted by FUL5, FUL10, FUL15, FUL20, FUL25, FUL30, FUL80, FUL85, FUL90 and FUL95 respectively. According to the solvent content in the two phases, FUL5-FUL30 can be considered simulated raffinate-phase samples, and FUL80-FUL95 can be considered simulated extract-phase samples.

DAO and FUL were placed in an extraction kettle according to a certain ratio of solvent to oil and stirred for 30 min under the conditions of 100 °C and 500 RPM. After standing for 1 h, the raffinate and extract phases were released from the discharge bottom valve and stirred well to obtain the real DAO raffinate- and extract-phase samples, which were respectively recorded as R-SL-x and E-SL-y (where x and y are 1–5).

### 2.3. Analysis of SARA Fraction Contents

In accordance with the method for separation of asphalt into four fractions (NB/SH/T0509-2010), which was further improved by patent CN102527088 A [32], the saturate and aromatic fractions in the sample were separated by column chromatography at -20 KPa, and the contents of saturate and aromatic fractions in the sample were recorded as $w_{saturates}$ and $w_{aromatics}$, respectively. Since the asphaltene fraction content in DAO was less than 1%, the contents of asphaltenes and resins fractions were summed as $w_{re\sin s+asphaltenes}$ (%), which was obtained by the subtraction method using Equation (1).

(1) $$w_{re\sin s+asphalenes}=(1-w_{saturates}-w_{aromatics})\times 100\%$$

where $w_{saturates}$ and $w_{aromatics}$ are the mass fractions of saturate and aromatic fractions in the samples, respectively.

### 2.4. Simulation and Calculation 

The geometries of saturate, aromatic and resin model molecules and FUL molecules were optimized by using the GGA-BLYP functionals of the DMol3 module in MaterisStudio software (MS2019 version), and the corresponding electrostatic potential (ESP) distributions were obtained. COSMO files for subsequent calculations were obtained using Tmolex2021. As shown in Figure 1a–c, saturate, aromatic and resin model molecules represented the saturate, aromatic, and resin fractions in DAO, respectively, and they were based on the G&D model [33], combined with the cyclic rings, aromatic rings, total ring number, and side-chain length of each component structural unit in *Petroleum refining engineering*, 4th edition, page 48 [34].

The adsorption energy of saturate, aromatic and resin model molecules and FUL molecules on the surface of FCP-Al_2_O_3_ (100) was calculated using the adsorption locator module in MS2019. The simulated annealing calculation parameters were set as 100,000 loading steps, 5 heating cycles, and 50,000 steps per cycle for structural optimization. The energy parameters were selected as COMPASS (Version 2.8) Forcefield and Charges of forcefield assigned. In addition, the electrostatic terms were selected as the summation method of Ewald, an accuracy of 0.0001 kcal/mol, and a buffer width of 0.5 A. The van der Waals terms were selected as the atom-based summation method, the truncation method of cubic spline, a cutoff distance of 15.5 A, a spline width of 1 A, and a buffer width of 0.5 A.

Interaction energies between the saturate, aromatic, and resin model molecules and the furfural molecules were calculated using COSMOtherm2021 (version: BP_TZVPD_FINE_21.ctd) at the BP-TZVP quantization level and corresponding parameters.

### 2.5. Determination of FUL Content

The schematic diagrams of the traditional vacuum distillation method and the improved vacuum distillation method for the determination of FUL content are shown in Figure 2a and Figure 2b respectively. When the traditional vacuum distillation method is used to analyze solvent content in the raffinate and extract phases of heavy oil, a single set of traditional vacuum distillation devices can only analyze one sample at a time; thus the efficiency is relatively low. Furthermore, it is difficult to maintain identical analysis conditions for each experiment, which affects the accuracy of the data. To overcome the shortcomings of the above method, in this study, the traditional vacuum distillation device was improved into a vacuum-drying oven. Besides, a low-temperature condenser was installed between the vacuum oven and the oil pump for solvent recovery, avoiding the corrosion of the sealing components of the oil pump caused by solvent vapor. Compared with the traditional vacuum distillation method, the improved vacuum distillation method, namely, the vacuum-oven method, can analyze multiple parallel samples at one time with higher efficiency. In addition, the analytical conditions can be kept consistent for each experiment, thus improving the accuracy of the data.

The detailed experimental procedure for the determination of furfural content by the vacuum-oven method is as follows: an empty weighing bottle (L 40 × Ф 25 mm) with constant weight after drying was weighed using a high-precision analytical balance (AB135-S/FACT METTLER TOLEDO, Zurich, Switzerland), and then the balance was adjusted to zero value. Secondly, the samples prepared in accordance with Section 2.2 were taken by a 2 mL pipette and added into these weighing bottles for the accurate weighing of the samples. Then the weighing bottles containing samples were transferred to an intelligent electric heating vacuum-drying oven (6090AB, Beijing Hennuo Lixing Technology Co., Ltd, Beijing, China) equipped with a rotary-vane vacuum pump (2XZ-4, Shanghai Double Goose Refrigeration Co., Ltd, Shanghai, China). After the set time, the weighing bottles were taken out while hot, immediately transferred to the desiccator (Ф 400 mm; containing 1 kg of silica gel self-indicator), and weighed after cooling to room temperature (see Figure 2b). The content of FUL in samples (*w*, %) was calculated by using Equation (2).

(2) $$w=\frac{m_{0}+m_{s}-m_{1}}{m_{s}}\times 100\%$$

where $m_{0}$ is the mass of the empty weighing bottle (g), $m_{s}$ is the mass of the sample (g), and $m_{1}$ is the mass of the weighing bottle containing sample that has been heated and vacuumed to remove FUL (g).

The accuracy was measured by the recovery of FUL in samples for the raffinate phase and the extract phase. The closer the recovery rate is to 100%, the higher the accuracy is. The recovery of FUL was calculated by using Equation (3).

(3) $$Re\mathrm{cov}ery=\frac{w}{w_{ture}}\times 100\%$$

where $w_{ture}$ is the true value of the content of FUL in the sample and w is the measured value of FUL content in the sample.

Precision was measured by the relative standard deviation (RSD) of multiple parallel determination values of FUL content in the sample [35]; the closer the RSD is to 0, the better the precision is. RSD was calculated by using Equation (4).

(4) $$RSD=\frac{SD}{\bar{w}}\times 100\%$$

where *SD* is the standard deviation of multiple parallel determination values of FUL content in the sample and $\bar{w}$ is the arithmetic mean of multiple parallel determination values of FUL content in the sample.

*SD* was calculated by using Equation (5).

(5) $$SD=\sqrt{\frac{\sum_{i=1}^{n}{\left(w_{i}-\bar{w}\right)}^{2}}{n-1}}$$

where *n* is the number of parallel determination experiments and $w_{i}$ is the measured value of FUL content in the i-th sample in n parallel experiments.

## 3. Results and Discussion

### 3.1. Effect of FUL on the Analysis of Contents of SARA Fractions

In order to investigate the effect of FUL on the analysis of contents of SARA fractions, the contents of SARA fractions in the simulated raffinate and extract phases were analyzed by elution chromatography, and the experimental phenomena were observed. In Figure 3a, the chromatographic bands after sample loading correspond to FUL0, FUL10, FUL20, FUL30, FUL80 and FUL90 from left to right. Compared with FUL0 (blank sample without furfural), the chromatographic bands of FUL10, FUL20 and FUL30 did not show marked variation, while the chromatographic bands of FUL80 and FUL90 moved downward significantly. Figure 3b shows the chromatographic bands after eluting the saturate fraction. Compared with FUL0, the chromatographic bands of FUL10, FUL20 and FUL30 did not change obviously, while the yellow chromatographic bands of FUL80 and FUL90 moved downward obviously. Figure 3c displays the chromatographic bands after eluting the aromatic fraction. Compared with FUL0, the corresponding chromatographic bands of FUL10, FUL20 and FUL30 did not change significantly, while the reddish-brown chromatographic bands of FUL80 and FUL90 moved downward obviously.

Figure 3d,e respectively show the eluates of the saturate and aromatic fraction corresponding to FUL0, FUL10, FUL20, FUL30, FUL80 and FUL90 from left to right. According to the color change of the eluate of the saturate fraction, compared with FUL0, the eluates of the saturate fraction corresponding to FUL10, FUL20 and FUL30 were colorless, while the eluates of the saturate fraction corresponding to FUL80 and FUL90 were slightly light yellow, indicating that the eluates of the saturate fraction of FUL80 and FUL90 were mixed with a small amount of aromatic fraction. Compared with FUL0, the eluates of the aromatic fraction corresponding to FUL10, FUL20 and FUL30 were bright yellow, while the eluates of the aromatic fraction corresponding to FUL80 and FUL90 were obviously reddish brown, indicating that some resin fraction was mixed in the eluates of the aromatic fraction corresponding to FUL80 and FUL90.

The contents of SARA fractions in the simulated two-phase samples are listed in Table 2. Compared with FUL0, the contents of the SARA fractions in FUL10, FUL20 and FUL30 did not change significantly. However, the contents of saturate and aromatic fractions in FUL80 and FUL90 increased, but the contents of resin and asphaltene fractions decreased significantly, again indicating that the saturate fraction in FUL80 and FUL90 contained a small amount of aromatic fraction and the aromatic fraction contained some resin fraction.

Based on the variation in the chromatographic bands, the color of the eluates of saturate and aromatic fractions, and the content data of SARA fractions mentioned above, the results show that the influence of FUL on the content analysis of SARA fractions for FUL10, FUL20 and FUL30 samples with low content of FUL was relatively small, while the influence of FUL on the content analysis of SARA fractions for FUL80 and FUL90 samples with high content of FUL was more significant.

### 3.2. Molecular Simulation of the Effect of FUL on the Analysis of SARA Fractions

In Section 3.1, the significant influence of the higher content of FUL in the sample on the content analysis of SARA is described from a macroscopic perspective by experimental means. To investigate the reasons, the adsorption of FUL molecules and saturate, aromatic, and resin model molecules onto the surface of FCP-Al_2_O_3_ and the interactions between FUL and saturate, aromatic, resin model molecules were analyzed from a microscopic perspective by molecular simulations, and the influence mechanism of furfural on saturates, aromatics, and resins was proposed.

#### 3.2.1. Adsorption of Saturates, Aromatics, and Resins and FUL onto the Surface of FCP-Al_2_O_3_

Elution chromatography is based on the adsorption difference between each component of the mixture and the adsorbent, realizing the separation of each component under the elution of the mobile phase. The sample-loading process, as an important step in the process of elution chromatography to separate SARAs in heavy oil, can be simulated by the dynamic process of adsorption and desorption of SARA model molecules and FUL molecules on the surface of a model of column packing. In this work, FUL, saturate, aromatic, and resin model molecules and FCP-Al_2_O_3_ were selected to carry out molecular simulation research on the interaction between these model molecules and FCP-Al_2_O_3_. Figure 4 represents optimized structures of saturate, aromatic, and resin model molecules and FUL adsorbed onto the surface of FCP-Al_2_O_3_, where the aromatic rings in aromatics and resins were close to the surface of FCP-Al_2_O_3_. The adsorption energies (Table 3) of saturate, aromatic, resin and FUL model molecules on the surface of FCP-Al_2_O_3_ were −400.35, −473.71, 599.99, and 496.15 kJ/mol, respectively. Therefore, the order of adsorption ability of model molecules and FUL on the surface of FCP-Al_2_O_3_ was saturates < aromatics < FUL < resins.

According to the mechanism of the acidic centers, the interactions between polycyclic aromatic hydrocarbons (PAHs) and the acidic sites on the adsorbent surface result in the adsorption of PAHs [36,37,38]. From the perspective of Lewis acid–base theory, resin and aromatic model molecules have the ability to donate electrons, and their π-electrons can interact with the Lewis acid center in FCP-Al_2_O_3_. The adsorption energies of saturate, aromatic and resin model molecules on the surface of FCP-Al_2_O_3_(100) were gradually enhanced, which is mainly attributed to resins and aromatics containing aromatic rings, and electron density on the aromatic rings in resins was higher than that in aromatics (See Figure 5), resulting in stronger interaction between the π-electrons on the aromatic ring in resins and the Lewis acid center than that between aromatics and the center. However, there are no aromatic rings in saturates, and van der Waals interaction was the dominant interaction between saturates and the Lewis acid center, so the adsorption energy of saturates on the surface of FCP-Al_2_O_3_(100) was the weakest.

#### 3.2.2. Interactions Between FUL and Saturate, Aromatic, and Resin Model Molecules

The σ-profiles of saturates, aromatics, resins and FUL are shown in Figure 6. The σ-profile of FUL had a wider distribution than that of the saturate, aromatic and resin model molecules, indicating that FUL was more polar than the saturate, aromatic and resin model molecules. In addition, the distribution width of the screening charge of saturate, aromatic and resin model molecules increased in sequence, indicating that the order of polarity for the model molecules was saturates < aromatics < resins. According to the principle of “like dissolves like”, unadsorbed furfural could have promoted the desorption of the saturate, aromatic and resin molecules which were adsorbed onto the surface of FCP-Al_2_O_3_ (the promotion effect is similar to elution, and the effect is enhanced in turn).

It can be seen from Table 4 that the interaction energy values between saturate, aromatic, resin molecules and FUL were −82.278, −88.197, and −104.56 kJ/mol respectively, indicating that the order of interaction between saturate, aromatic, resin model molecules and FUL was saturates < aromatics < resins, which is consistent with the order of polarity of saturate, aromatic, and resin model molecules. The reason for the increasing interaction between saturate, aromatic, resin and FUL molecules is mainly that the electronic cloud density of resins is greater than that of aromatics, so the interaction between the electron-deficient H atom of the five-membered ring in FUL and the π-electron of the aromatic ring in resins was greater than that between aromatics and FUL; besides, there are no aromatic rings in saturates, so the interaction between aromatics and FUL was greater than that between saturates and FUL. Based on the difference in the interaction between FUL and the model molecules, the promotion effect of unadsorbed FUL on the desorption of the saturate, aromatic, resin model molecules adsorbed onto the adsorbent was gradually enhanced.

#### 3.2.3. Influence Mechanism of FUL on the Analysis of SARA Fractions

In order to understand the influence of FUL on the analysis of SARA fractions in the raffinate and extract phases of DAO more clearly, a mechanism is proposed as shown in Figure 7. When the sample did not contain FUL, the ability of the saturate fraction, aromatic fraction and resin fraction to adsorb onto the surface of FCP-Al_2_O_3_ was sequentially enhanced during the sample-loading process, resulting in the chromatographic bands of resins, aromatics and saturates being in order from top to bottom. During the elution process, the DAO samples were separated into saturate, aromatic and resin fractions by using eluents with sequentially increasing polarity (such as n-heptane, toluene, and toluene–ethanol). When the content of FUL in the sample was higher, during the sample-loading process, the ability of FUL to adsorb onto the surface of FCP-Al_2_O_3_ was stronger than that of aromatics and saturates. Therefore, FUL was preferentially adsorbed onto the surface of FCP-Al_2_O_3_ and occupied the adsorption sites that originally belonged to aromatics and saturates, which made the chromatographic bands of aromatics and saturates move downward as a whole. On the other hand, the interaction between unadsorbed polar solvent FUL and saturate, aromatic and resin molecules was sequentially enhanced, indicating that the promotion effect of unadsorbed FUL on the desorption of the saturate, aromatic and resin model molecules adsorbed onto the adsorbent was enhanced in turn, resulting in a significant downward shift of the chromatographic bands of resins and aromatics. In summary, the comprehensive influence of a large amount of FUL on the adsorption and desorption of saturates, aromatics and resins on the surface of FCP-Al_2_O_3_ eventually led to some aromatics being eluted earlier when eluting saturates with n-heptane and to some resins being eluted earlier when eluting aromatics with toluene.

### 3.3. Optimization and Testing of Analysis Method for FUL

The results of the determination of the contents of SARA fractions in the simulated raffinate and extract phases containing FUL indicate that higher FUL content in the sample did have influence on the analysis of SARA fractions, and the mechanism of influence of furfural on the analysis of SARA contents was revealed by molecular simulation. On one hand, the production process required the control of furfural content in the product or intermediate product. On the other hand, the analysis of SARA contents in the oil required that the solvent be removed. Therefore, it is necessary to provide an accurate method for the analysis of furfural content. The improved vacuum-oven method is described in the Section 2 (Figure 2). In order to verify the reliability of the method, first, the analytical conditions were optimized, and then the precision and accuracy of the method were investigated.

#### 3.3.1. Optimization of Analytical Conditions

The effects of sample mass, the set time of the oven for processing the sample, the set vacuum degree, and the set temperature of the oven on the furfural recovery rate for FUL10 are listed in Figure 8 a, b, c, and d respectively. The furfural recovery rate for FUL10 gradually decreased with the increase in sample mass and increased with the increase in set time, set vacuum degree, and set temperature of the oven. In addition, the furfural recovery rates for FUL10 corresponding to a sample mass of 0.8g, a set time of 60min, a set vacuum degree of 93 KPa, and a set temperature of 105 °C were 100.06%, 100.11%, 100.28%, and 99.879%, respectively. Accordingly, it is suitable to choose the above conditions as the conditions for furfural content analysis in the simulated raffinate phase.

On the premise of not affecting the accuracy and precision of the experimental results and in order to improve the operability and convenience of the analytical experimental process, the sample mass and vacuum degree of the conditions for furfural content analysis in the simulated extract phase were kept consistent with those of the simulated raffinate phase. Therefore, it was only necessary to study the effects of set time and set temperature of the oven on the recovery of furfural from the simulated extract phase. The effects of set time and set temperature on the recovery of furfural from the simulated raffinate phase are represented in Figure 9, from which it can be seen that the recovery of furfural increased with the increase in time and set temperature. Besides, when the sample mass was 0.8 g and the set vacuum degree was 93 KPa, under the conditions of a set time of 50 min and a set temperature of 75 °C, the furfural recovery rate of FUL90 was closer to 100%. Consequently, it is more appropriate to select the above conditions as the conditions for furfural content analysis in the simulated extract phase.

#### 3.3.2. Accuracy and Precision of Vacuum-Oven Method

Under the optimized analytical conditions, the precision and accuracy in the determination of furfural content in the simulated raffinate phase are listed in Table 5. Obviously, the RSD of the measured value of furfural content in FUL5-30 was less than 1%, and the recovery of furfural was in the range of 99.9–100.3%. The RSD of the measured value of furfural content in FUL80-95 was less than 0.5%, and the recovery of furfural was in the range of 99.7–100.5%. This shows that the vacuum-oven method has good precision and accuracy in the determination of furfural content in simulated raffinate and extract phases.

The results of the determination of furfural content in real DAO raffinate- and extract-phase samples are summarized in Table 6. The RSDs of the measured values of furfural content in R-SL-1, R-SL-2, R-SL-3, R-SL-4, and R-SL-5 were 0.47%, 0.36%, 0.34%, 0.48%, and 0.92%, respectively. Similarly, the RSDs of the measured values of furfural content in E-SL-1, E-SL-2, E-SL-3, E-SL-4 and E-SL-5 were 0.11%, 0.06%, 0.08%, 0.06%, and 0.02%, respectively. Thus, it is now clear that the vacuum-oven method also has good precision in the determination of furfural content in real DAO raffinate- and extract-phase samples.

## 4. Conclusions

The analysis of SARA fractions revealed that the chromatographic bands, the colors of the eluates and the contents of SARA fractions were less influenced by FUL when its content in the sample was low. In contrast, the content analysis of SARA fractions was more significantly affected by the presence of furfural at higher concentrations in the sample. During the process of sample loading, certain chromatographic bands of resins and aromatics significantly shifted downward: certain aromatics were eluted earlier when saturates were eluted with n-heptane, and certain resins were eluted earlier during the elution of aromatics with toluene. The results of molecular simulations indicate that the adsorption energy of model molecules (saturates, aromatics, resins and FUL) on the surface of FCP-Al_2_O_3_ followed the order: saturates < aromatics < FUL < resins. Furthermore, the interaction energy between FUL and the model molecules was ranked as follows: saturates < aromatics < resins. During the process of sample loading, FUL preferentially adsorbs onto the surface of FCP-Al_2_O_3_. Concurrently, some unadsorbed furfural promotes the desorption of saturate, aromatic and resin molecules that were already adsorbed onto the surface of FCP-Al_2_O_3_ (this promotion effect is similar to elution, and the effect is enhanced in turn). Consequently, certain chromatographic bands of resins and aromatics experience a significant downward shift, resulting in the earlier elution of these compounds. Hence, the influence of FUL on the analysis of SARA fractions could be better explained by the results of molecular simulations. Furthermore, the optimized analytical conditions for the determination of furfural using the vacuum-oven method are a sample mass of 0.8 g, a set time of 60 min, a vacuum degree of 93 KPa, and a temperature of 105 °C for the simulated raffinate phase and a sample mass of 0.8 g, a set time of 50 min, a vacuum degree of 93 KPa, and a temperature of 75 °C for the simulated extract phase. Under optimized conditions, the vacuum-oven method not only has good precision (RSD < 1%) and accuracy (recovery: 99.7–100.5%) in the determination of furfural content in simulated raffinate- and extract-phase samples but also has good precision (RSD < 1%) on real raffinate- and extract-phase samples.

## Figures and Tables

**Figure 1:**
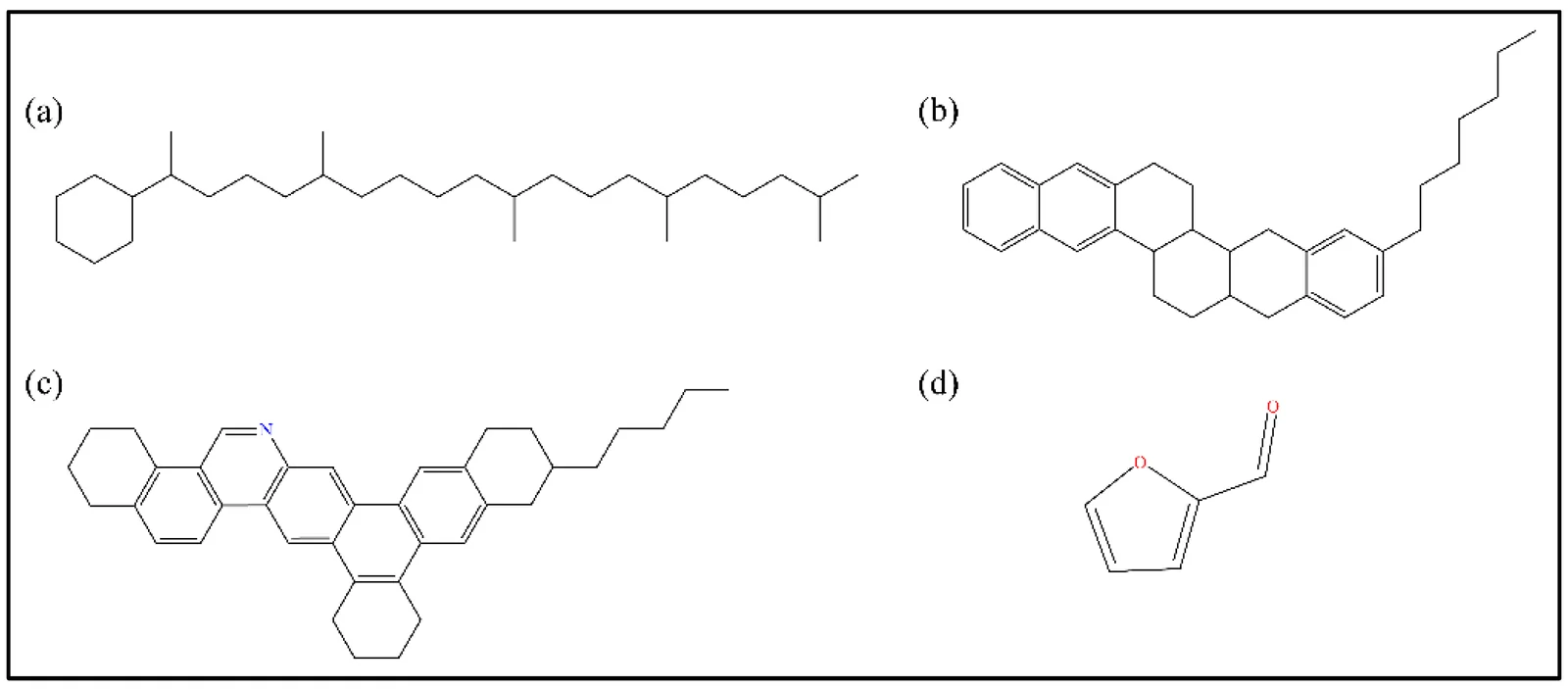
Molecular structures of (**a**) saturate, (**b**) aromatic, and (**c**) resin model molecules in DAO and (**d**) FUL.

**Figure 2:**
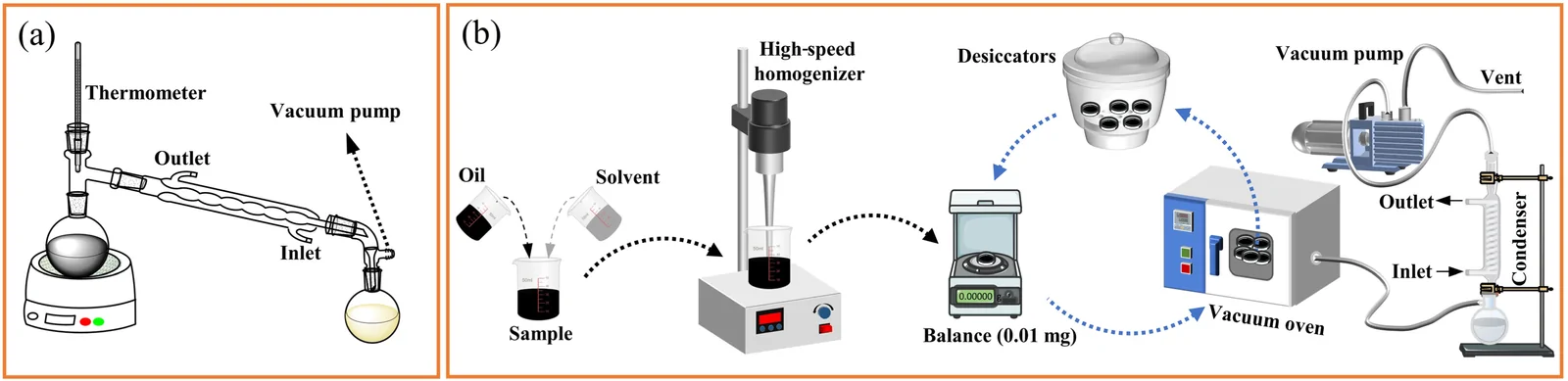
Schematic diagrams for determination of FUL in samples by (**a**) the traditional vacuum distillation method and (**b**) the improved vacuum distillation method.

**Figure 3:**
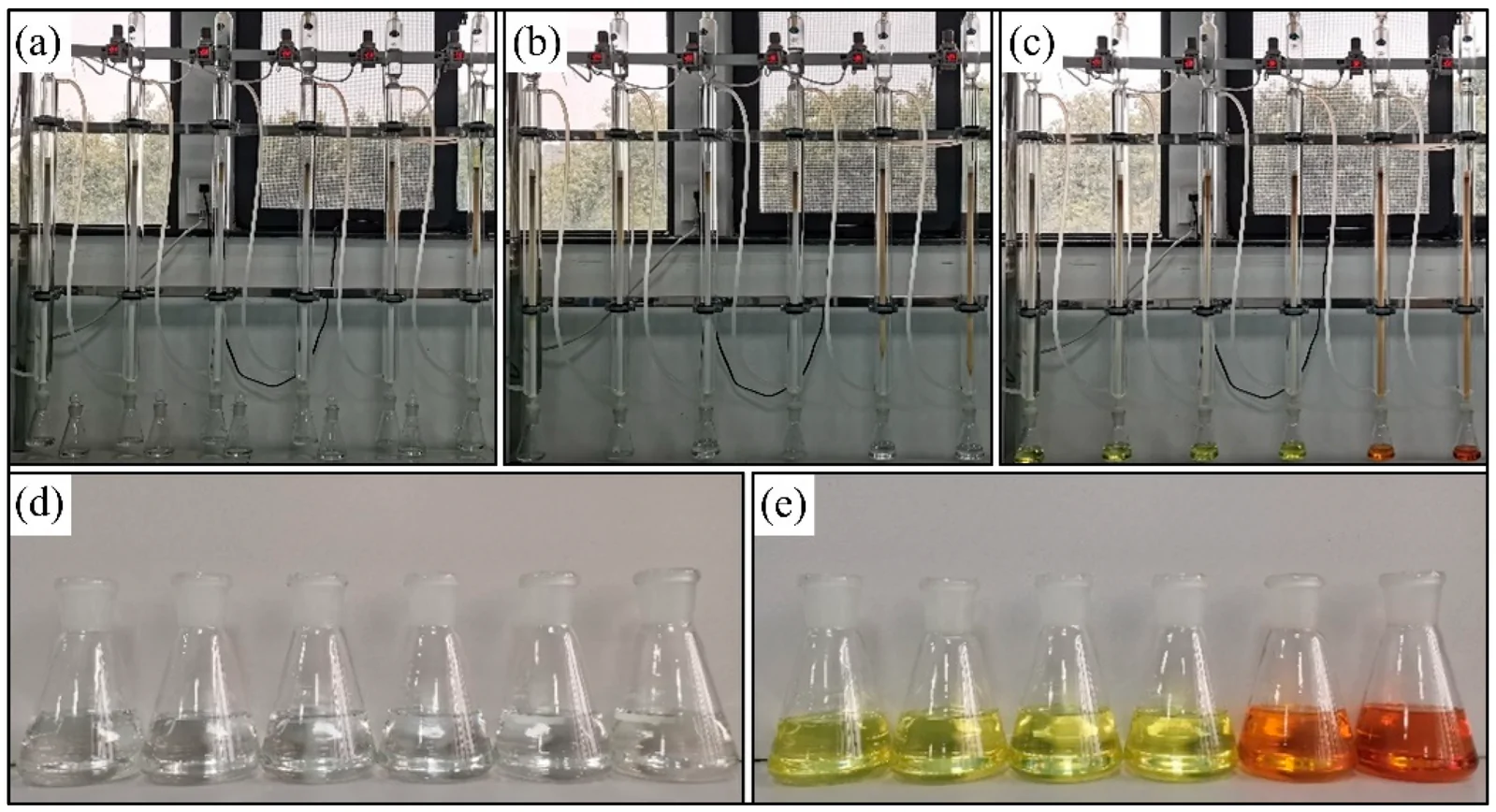
Experimental phenomena in the content analysis of SARA fractions in the simulated raffinate and extract phases. (**a**) Chromatographic bands after sample loading, (**b**) Chromatographic bands after eluting saturate fraction. (**c**) Chromatographic bands after eluting aromatic fraction. (**d**) Eluates of saturate fraction. (**e**) Eluates of aromatic fraction.

**Figure 4:**
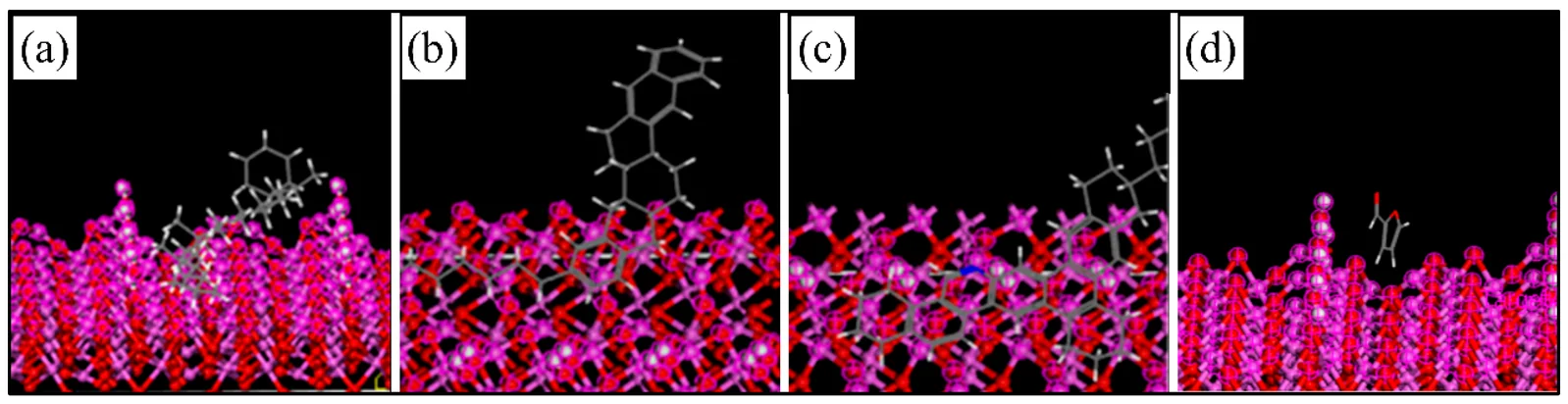
Optimization structures for adsorption of (**a**) saturates, (**b**) aromatics, (**c**) resins, and (**d**) FUL on the surface of Al_2_O_3_(100).

**Figure 5:**
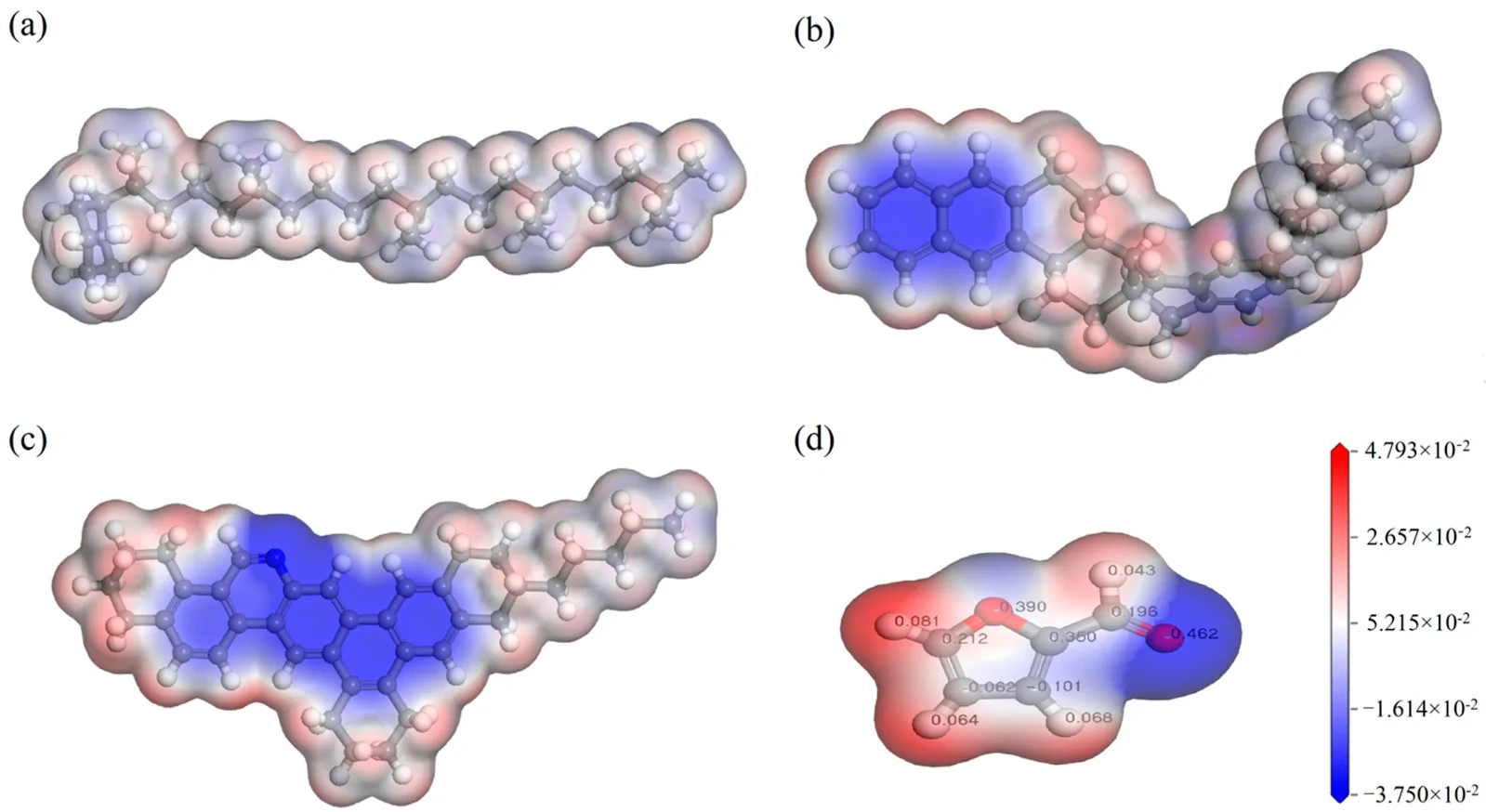
ESPs of (**a**) saturates, (**b**) aromatics, (**c**) resins, and (**d**) FUL.

**Figure 6:**
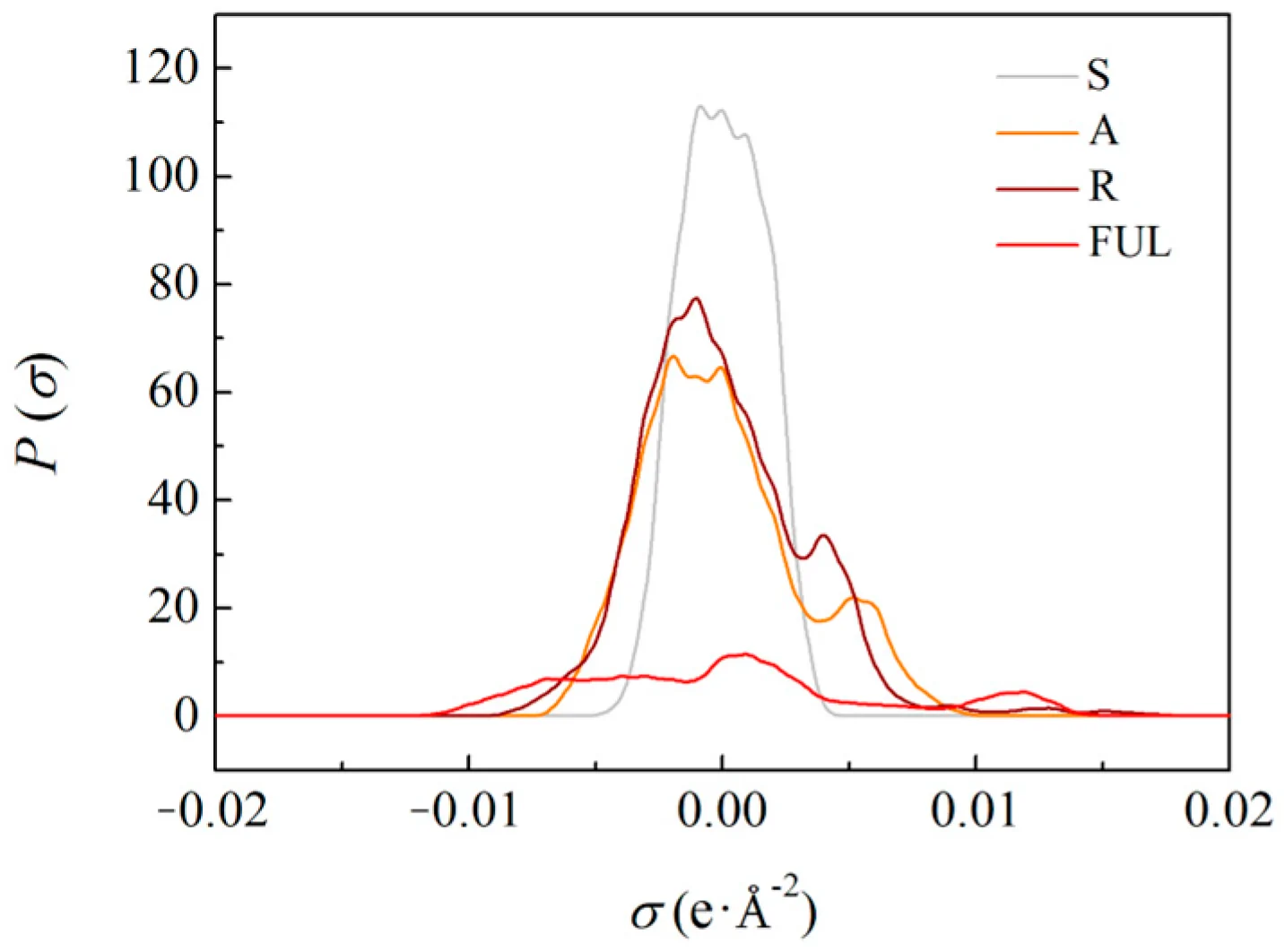
σ-Profiles of model molecules of saturates, aromatics, resins and FUL.

**Figure 7:**
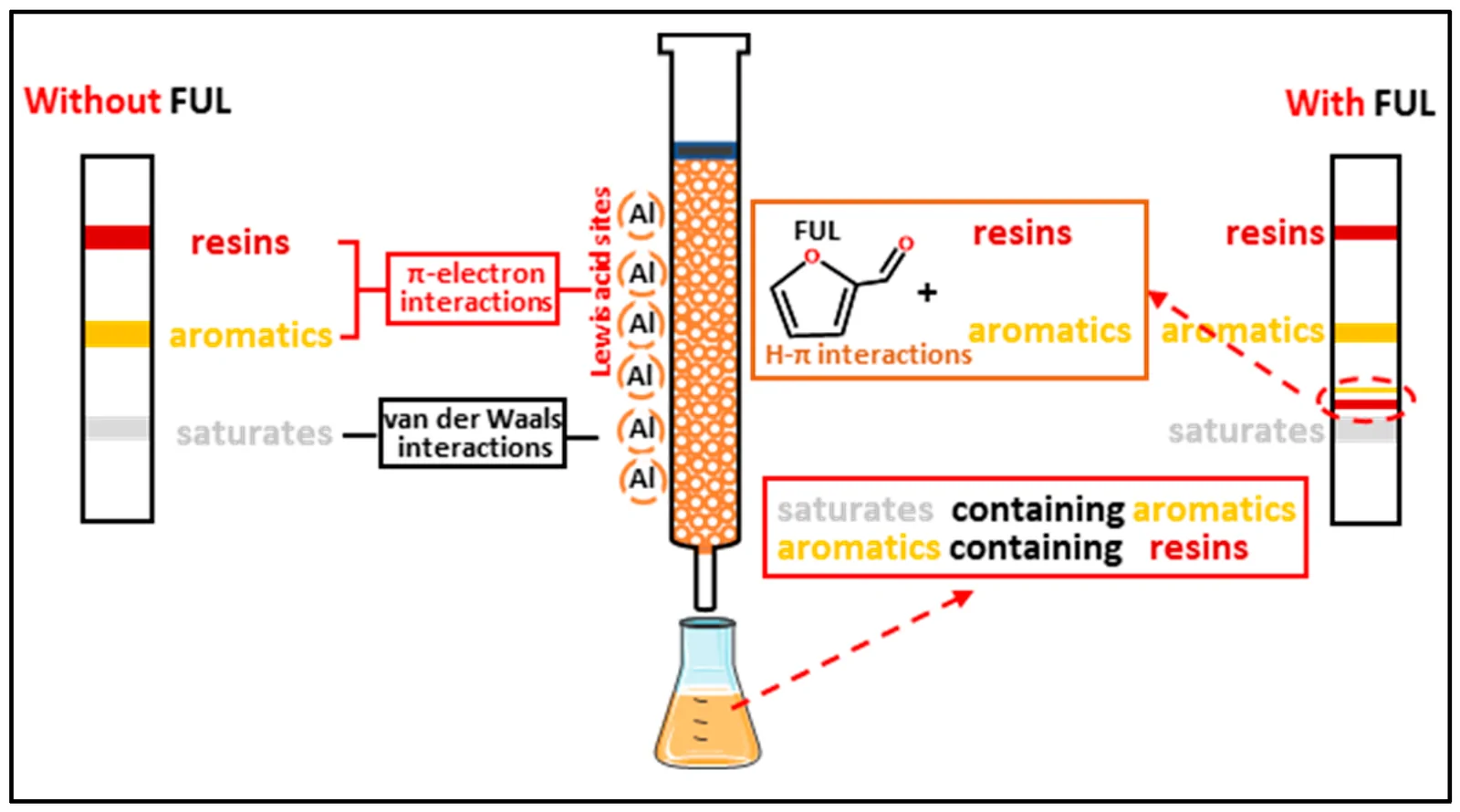
Influence mechanism of FUL on the analysis of SARA fractions in DAO by EC.

**Figure 8:**
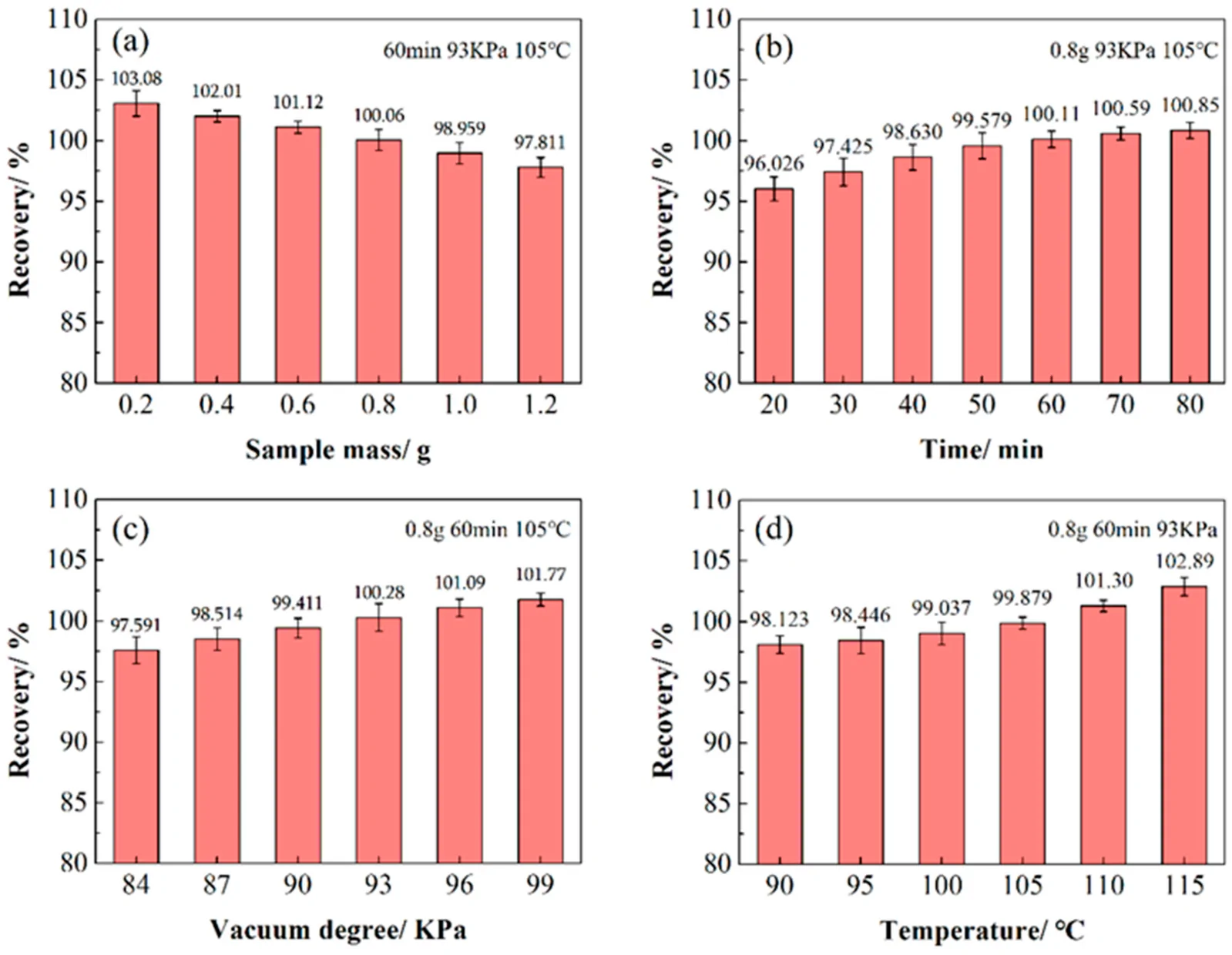
Influences of analysis conditions on furfural recovery from FUL10: (**a**) sample mass, (**b**) time, (**c**) vacuum degree, and (**d**) temperature.

**Figure 9:**
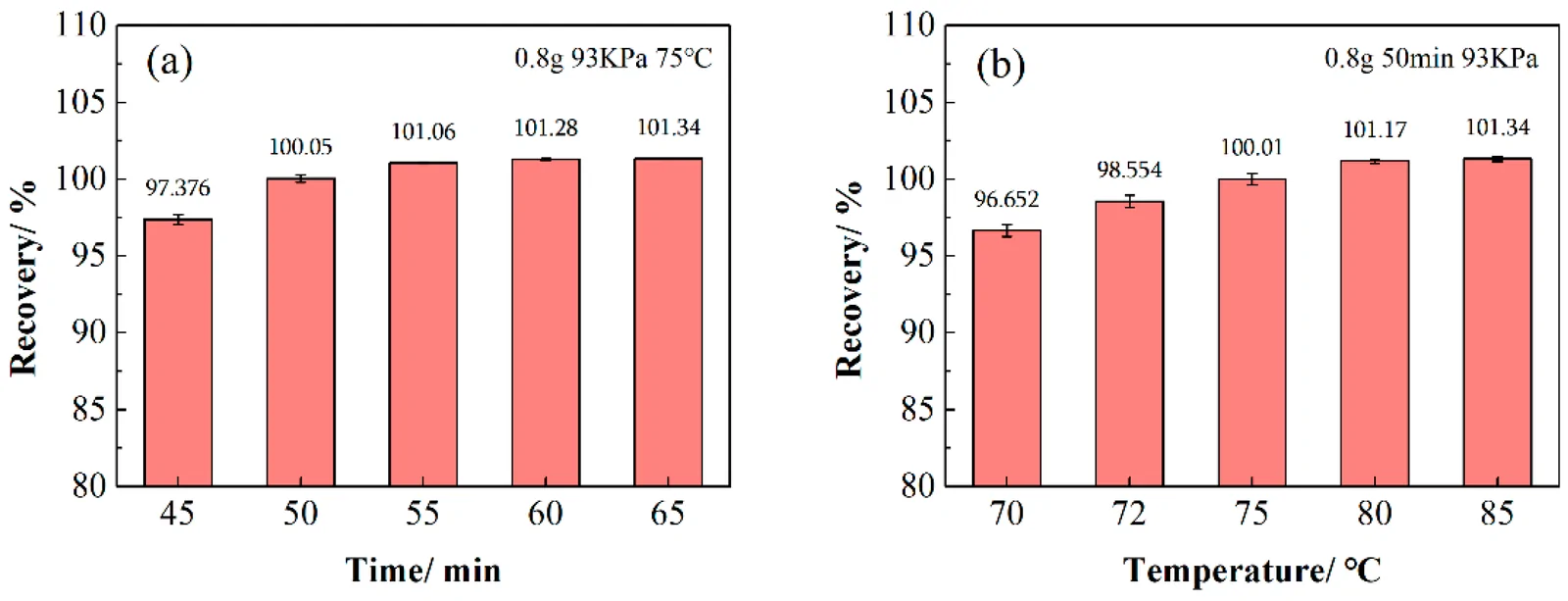
Influences of analysis conditions on the furfural recovery from FUL-90: (**a**) time and (**b**) temperature.

**Table 1:** Properties of DAO and HPO.

	DAO	HPO
*ρ*, kg·m^−3^	922.4 ± 0.4 (50 °C)	866.2 ± 0.3 (25 °C)
*v*, mm^2^·s^−1^	6174.0 ± 6.5 (50 °C)	25.71 ± 0.02 (25 °C)
*w_saturates_*, %	28.868 ± 0.165	97.256 ± 0.215
*w_aromatics_*, %	44.668 ± 0.615	0.569 ± 0.471
*w_resins+asphaltenes_*, %	26.464 ± 0.780	2.175 ± 0.686

**Table 2:** Contents of SARA fractions in samples in simulated raffinate and extract phases.

Samples	The Contents of SARA Fractions,%		
	*w* *_saturates_*	*w* *_aromatics_*	*w* *_resins+asphaltenes_*
FUL0	80.721 ± 0.348	8.4790 ± 0.5148	10.800 ± 0.863
FUL10	80.125 ± 0.485	8.7199 ± 0.4268	11.155 ± 0.912
FUL20	80.662 ± 0.214	8.7689 ± 0.5194	10.569 ± 0.733
FUL30	80.236 ± 0.314	9.0033 ± 0.5168	10.761 ± 0.831
FUL80	81.153 ± 0.257	10.344 ± 0.457	8.5030± 0.7142
FUL90	82.390 ± 0.371	12.392 ± 0.505	5.2180 ± 0.8754

**Table 3:** Adsorption energy of saturate, aromatic, resin and FUL model molecules on the surface of FCP-Al_2_O_3_(100), kJ/mol.

Saturates–Al_2_O_3_	Aromatics–Al_2_O_3_	Resins–Al_2_O_3_	FUL-Al_2_O_3_
−400.35	−473.71	−599.99	−496.15

**Table 4:** Interaction energy between saturates, aromatics, resins and FUL, kJ/mol.

Saturates–FUL	Aromatics–FUL	Resins–FUL
−82.278	−88.197	−104.56

**Table 5:** Precision and accuracy in determination of FUL content in the simulated raffinate and extract phases.

Sample	*w*_ture_ (%)	*w* (%, n = 5)					$\bar{w}$ (%)	RSD (%)	Recovery (%)
		1	2	3	4	5			
FUL5	5.0061	5.0160	5.0555	5.0653	4.9886	4.9550	5.0161 ± 0.0460	0.92	100.20
FUL10	10.006	10.010	9.9197	9.9760	10.159	10.098	10.033 ± 0.096	0.95	100.27
FUL15	15.004	15.136	15.111	15.065	15.011	14.921	15.049 ± 0.086	0.57	100.30
FUL20	20.013	20.035	19.959	20.025	20.011	19.981	20.002 ± 0.032	0.16	99.946
FUL25	25.009	25.056	25.073	24.972	24.990	24.880	24.994 ± 0.077	0.31	99.941
FUL30	30.000	29.934	30.083	30.090	29.956	30.000	30.013 ± 0.072	0.24	100.04
FUL80	79.999	79.756	79.945	80.159	80.204	80.357	80.084 ± 0.235	0.29	100.11
FUL85	84.998	84.628	85.287	84.733	85.461	84.850	84.992 ± 0.363	0.43	99.993
FUL90	90.009	89.708	89.632	90.050	89.719	89.868	89.795 ± 0.166	0.18	99.763
FUL95	94.993	95.535	95.520	95.131	95.469	95.618	95.455 ± 0.189	0.20	100.49

**Table 6:** Determination of FUL content in real DAO raffinate and extract phases.

Sample	*w* (%, n = 5)					$\bar{w}$ (%)	RSD (%)
	1	2	3	4	5		
R-SL-1	17.893	18.080	18.072	18.084	18.095	18.045 ± 0.085	0.47
E-SL-1	88.353	88.211	88.433	88.457	88.324	88.356 ± 0.098	0.11
R-SL-2	13.382	13.289	13.269	13.261	13.309	13.302 ± 0.048	0.36
E-SL-2	91.046	91.044	91.009	91.132	91.116	91.069 ± 0.052	0.06
R-SL-3	15.379	15.413	15.320	15.355	15.278	15.349 ± 0.052	0.34
E-SL-3	93.365	93.259	93.294	93.327	93.441	93.337 ± 0.070	0.08
R-SL-4	18.632	18.673	18.729	18.500	18.562	18.619 ± 0.090	0.48
E-SL-4	93.668	93.792	93.774	93.801	93.795	93.766 ± 0.056	0.06
R-SL-5	9.6711	9.8992	9.8241	9.8848	9.8119	9.8182 ± 0.0904	0.92
E-SL-5	94.599	94.564	94.596	94.597	94.584	94.588 ± 0.015	0.02

## Data Availability

The original contributions presented in this study are included in the article. Further inquiries can be directed to the corresponding author.
